# The Ashodaya PrEP project: Lessons and implications for scaling up PrEP from a community-led demonstration project among female sex workers in Mysore, India

**DOI:** 10.1080/17441692.2020.1724316

**Published:** 2020-02-18

**Authors:** Sushena Reza-Paul, Lisa Lazarus, Raviprakash Maiya, Partha Haldar, B. B. Rewari, M. S. Venugopal, Syed Hafeez Ur Rahman, K. T. Venukumar, Manjula Ramaiah, Akram Pasha, Mukta Sharma, Richard Steen, Robert Lorway

**Affiliations:** a Centre for Global Public Health, Rady Faculty of Health Sciences, University of Manitoba, Winnipeg, Canada; b Ashodaya Samithi, Mysuru, India; c Centre for Community Medicine, All India Institute of Medical Sciences, New Delhi, India; d World Health Organization, SEARO; e Department of Public Health, Erasmus MC, University Medical Centre Rotterdam, Rotterdam, Netherlands

**Keywords:** PrEP, sex work, HIV prevention, community-led, demonstration project

## Abstract

To inform PrEP roll out, Ashodaya Samithi, a sex workers’ collective, conducted a community-led prospective demonstration project among female sex workers in Mysore and Mandya, India. Following a community preparedness phase and pre-screening, participants were recruited for clinical screening and enrolment, provided PrEP as part of combination HIV prevention, and followed for 16 months. Adherence was measured by self-reported pill intake and by tenofovir blood level testing among a subset of participants. Of the 647 participants enrolled, 640 completed follow-up. Condom use remained stable and no HIV seroconversions occurred. Self-reported daily PrEP intake over the last month was 97.97% at the end of the study. Tenofovir blood levels >40 ng/mL (consistent with steady state dosing) were detected among 80% (*n* = 68/85) and 90.48% (*n* = 76/84) of participants at month 3 and 6, respectively. Our study holds important insights for rolling out PrEP in community settings as part of targeted HIV prevention interventions.

## Introduction

Pre-exposure prophylaxis (PrEP) has emerged as a relatively new biomedical HIV prevention option. Efficacy has been well established in clinical trials (see for example, Okwundu, Uthman, & Okoromah, 2012), however results have varied among participant groups. Trials have shown efficacy among men who have sex with men (Grant et al., 2014; Liu et al., 2014), people who inject drugs (Choopanya et al., 2013), and serodiscordant couples (Baeten et al., 2012), whereas two trials among women were terminated due to inefficacy resulting from low adherence (Marrazzo et al., 2015; Van Damme et al., 2012). Women in the FEM-PrEP and VOICE trials described a number of challenges to adherence, including stigma, risk perception, and difficulty with clinic attendance (Corneli et al., 2016; van der Straten et al., 2014). Research has also pointed to lower ‘forgiveness’ of missed doses in women due to relatively lower concentrations of PrEP in vaginal tissue, compared to rectal tissue, with women requiring a ‘near perfect’ adherence to reach protective therapeutic levels (Cottrell et al., 2016).

In 2014, the World Health Organization (WHO) released a strong recommendation for governments to consider adding PrEP to their combination prevention strategies for men who have sex with men (World Health Organization, 2014). As of 2015, this recommendation was expanded to promote offering PrEP to all individuals at ‘substantial risk’ of HIV infection as an option in comprehensive HIV prevention services (World Health Organization, 2015). However, low adherence levels in clinical trials highlight the need for more research regarding PrEP use among women, including sex workers who were excluded from those studies (Cáceres et al., 2015; Shannon et al., 2018).

Studies have indicated an acceptability and willingness to take PrEP among sex workers (Eisingerich et al., 2012), with some studies documenting high interest in taking PrEP despite limited knowledge (Restar et al., 2017; Ye et al., 2014). A study exploring the acceptability of PrEP among sex workers in Baltimore indicated high self-efficacy for daily oral adherence (79%), with 78% of participants indicating an interest in using PrEP even with messaging that continued condom use was still a necessity (Peitzmeier et al., 2017). Our own feasibility study among sex workers belonging to the Ashodaya Samithi collective in Mysore, India documented high interest, with 95% of participants expresssing an interest in taking PrEP and 99% stating that they would also continue to use condoms while taking PrEP (Reza-Paul et al., 2016).

Demonstration projects now serve to offer insights into the delivery and scale up of PrEP, with early findings starting to emerge. Whereas sex workers were notably left out of clinical trials, they have been a key group in demonstration projects. According to the AIDS Vaccine Advocacy Coalition (AVAC), there are 18 ongoing or completed projects with sex workers, 15 of which include women sex workers (AVAC, 2018). While results from these projects are only beginning to be published, initial findings from some of these studies have shown low levels of retention over the course of the project. For example, the TAPS Demonstration Project was an observational cohort study that offered early antiretroviral therapy (ART) or PrEP to female sex workers in South Africa (Eakle et al., 2017). Wits Reproductive Health and HIV Institute (Wits RHI), a prominent research institute in South Africa, led the TAPS project through their existing sex work programme, which is run by nurses, community health workers, and peer outreach workers at two urban clinic sites providing primary health care services. The study found a PrEP uptake of 98%, with only 22% of participants remaining on PrEP at 12 months (Eakle et al., 2017).

SAPPH-Ire was a cluster-randomized trial that offered combination HIV prevention and treatment within the existing national sex work programme in Zimbabwe (Cowan et al., 2018). Cluster areas surrounding sex work clinics were enrolled in matched pairs to receive usual care (sexual health services supported by peer outreach workers, including HIV testing, referral for ART, and health education) or an intervention that supported additional regular HIV testing, on-site initiation of ART or PrEP, and intensified community mobilisation. PrEP uptake was 38% among women who tested negative for HIV. Retention was found to be low, with women taking PrEP for an average of just over four months. The SAPPH-Ire researchers state that the evidence for the effectiveness of PrEP in women was less known at the time of the roll out of the study and that the number of peer outreach workers supporting communities were low in both arms of the trial, which they conclude points to the need for effective adherence support strategies (Cowan et al., 2018).

In India, two demonstration projects led by sex worker organisations, the Durbar Mahila Samanwaya Committee (DMSC) in Kolkata, West Bengal (Ghose, Swendeman, George, & Chowdhury, 2008; Jana & Singh, 1995; Jana, Basu, Rotheram-Borus, & Newman, 2004) and Ashodaya Samithi in Mysore-Mandya, Karnataka (Argento et al., 2011; Reza-Paul et al., 2008; Reza-Paul et al., 2012), have recently been completed. India has the third largest HIV epidemic in the world with an estimated 2.14 million people living with HIV and 87,580 new infections in 2017 (National AIDS Control Organization & ICMR-National Institute of Medical Statistics, 2018). Higher prevalence is seen in key population groups, such as female sex workers, men who have sex with men, and people who inject drugs. While national HIV prevalence has decreased among sex workers from 10.33% in 2003 to 1.56% in 2017, prevalence is higher in certain states such as Karnataka, where prevalence remains above the national average at 3.33% (National AIDS Control Organization, 2017).

Targeted interventions (TIs) in India, led by the National AIDS Control Organization (NACO), have led to sharp declines in HIV incidence over the last decade. These TIs have largely focused on promoting consistent condom use among sex workers and their clients, with 90% of female sex workers reporting using condoms with new clients (Mitchell et al., 2016; Reza-Paul et al., 2008). However, condom use continues to remain low with regular partners, such as husbands and boyfriends (Deering et al., 2011; Fehrenbacher et al., 2018; Isac et al., 2017). The results from various surveys in Mysore show that while reported condom use is high in the last sex act with occasional clients, consistent condom use with all clients and partners continues to be a challenge (Reza-Paul et al., 2008; Reza-Paul et al., 2019). PrEP now presents a potential means of addressing this gap (Mayer, Chandhiok, & Thomas, 2016). Mathematical modelling parameterised to the HIV epidemic in Bangalore found that providing PrEP to female sex workers or men who have sex with men could substantially reduce HIV incidence among the prioritised group, with the greatest population-level impact seen when sex workers were prioritised (Mitchell et al., 2016). In qualitative research exploring the willingness to implement PrEP, policymakers and health care professionals in India expressed that they felt that current HIV prevention efforts were insufficient, but that local data was needed to make the case for allocating funding towards PrEP (Wheelock et al., 2012).

Much has been written about community empowerment approaches to HIV prevention among sex workers (Kerrigan et al., 2015). Communities, however, have typically not played key roles in clinical and biomedical interventions. The two demonstration project sites in India were selected due to their expertise in community-led interventions. In this paper, we present findings from the Ashodaya PrEP Demonstration Project, which provides a novel example of a community-led approach to providing PrEP through a well-established sex worker-led organisation. The study aimed to understand PrEP implementation, including delivery, retention, adherence, and follow-up, among female sex workers (FSWs), as well as strategies for integrating PrEP within the existing prevention programme.

## Methods

### Population and study setting

The study population was comprised of women who self-identified as sex workers and/or who received goods or money in exchange for sex in the past three months, were aged 18 or above, and worked within the districts of Mysore and Mandya, in South India.

In 2004, Mysore was the first intervention site supported through the Bill & Melinda Gates’ Avahan Initiative, implemented directly by the University of Manitoba and scaled up to the neighbouring city of Mandya within a year. This site was initiated with a very high degree of community involvement. Over the years, the sex work communities came together and formed their own organisation known as Ashodaya Samithi, which has a current membership of over 8000 female, male, and hijra/transgender sex workers across four districts in the state of Karnataka. Ashodaya has been implementing numerous health programmes (such as condom promotion, screening and treatment for STIs and HIV, reproductive health services, and tuberculosis screening and treatment), preventing gender-based violence, and promoting social entitlement programmes by adopting community-led approaches. This enabled Ashodaya sex workers to achieve reductions in HIV prevalence and STIs and reduce violence among sex workers by using community-led approaches (Reza-Paul et al., 2008, 2012). As a result of its expertise in community-led interventions, Ashodaya has been recognised globally as a community-based research and learning site to disseminate and adapt its core principles and innovations to other sex work organisations.

### Study design

The project follows a prospective observational study design with a single arm. PrEP was implemented as part of a combined HIV prevention strategy and provided free of charge to HIV-negative FSWs following a screening process and informed consent.

### Sample size and inclusion criteria

The study aimed to enrol a total of 600 FSWs into the demonstration project. To enrol this many women, peer outreach workers aimed to reach most of the approximately 3000 FSWs enrolled with Ashodaya Samithi in the two districts of Mandya and Mysore.

At enrolment, participants had to: self-identify as a sex worker and currently be engaged in sex work; be 18 years or older; express an interest in and willingness to take PrEP; test negative for HIV; show no clinical signs of acute HIV infection; test negative for Hepatitis B; have a creatinine clearance ≥  = 60 ml/min (Cockcroft-Gault formula) upon screening; test negative for pregnancy (urine test); live within the operational area of the project site with no plans to move in the next 16 months; and express a willingness to avoid pregnancy during the 16-month study period.

### Intervention and recruitment

Participants were recruited through the existing system of peer outreach workers. The intervention was designed to be integrated into Ashodaya’s existing TI programme and therefore drew on the organisation’s community-owned health services and outreach and community mobilisation supports. Community preparedness for the project was ongoing, starting prior to a formal feasibility study. Peer outreach workers played a key role in disseminating information about PrEP to their networks. The findings from the feasibility study showed overwhelming support for moving forward with the demonstration project in terms of both acceptability and willingness among FSWs to use PrEP (Reza-Paul et al., 2016).

Under Ashodaya’s existing TI programme, peer outreach workers make contact with members of their sex work networks in the field (at least once fortnightly) to distribute condoms, answer questions, or make service referrals, with clinic visits occurring once per three months. This was done using a microplanning tool that ensures that the peer educators are meeting with the members of their network at a regular (fortnightly) interval for outreach (Bhattacharjee et al., 2018). As the demonstration project was integrated alongside the TI, it took advantage of this system of peer outreach workers to provide: (1) information about PrEP through individual or group discussions, clarifying concerns if any and referral to the community clinic for possible enrolment; (2) individualised PrEP dispensation plans; (3) support and monitoring for adherence, side effects, and condom provision; (4) use of Ashodaya’s community clinic services (either outreach or static clinic); and (5) use of Ashodaya’s community-based laboratory for pregnancy, HIV, and syphilis screening.

Peer outreach workers identified potential participants during their field visits and upon agreement, accompanied them to the Ashodaya clinic. Some women came directly to the clinic themselves. At the clinic, the first point of contact was a trained community counsellor. After a discussion with the community counsellor, if participants expressed interest, they were referred to the clinic counsellor for a pre-screening, which included counselling and informed consent. The physician then conducted a physical examination, and referred the participant to the lab for screening tests. Pregnancy, HIV, and syphilis testing were done in Ashodaya’s community-based lab. A private local lab chosen by the community was used for all other screening tests. Peer outreach workers accompanied potential participants to the lab. Early on, Ashodaya leadership worked with the lab personnel to ensure same day test results in order to minimise loss to follow-up between screening and PrEP initiation. Once the lab sent the results to the physician, they were shared with the potential participant, who once again was given the chance to withdraw from the study. If the participant again provided consent, the clinic counsellor would then administer the baseline questionnaire. If a participant chose to withdraw from the project, a short questionnaire was conducted to collect information on reasons for their refusal to participate. At the time of enrolment, a clinical history, general clinical examiniation, STI screening using an enhanced syndromic case management (SCM), approach, as well as syphilis screening using RPR were done. Counselling on how to use PrEP and possible side effects and their management was also provided. In most cases, the lab results were provided within two hours’ time. This was negotaited with the lab as a means of streamlining the process by providing same day enrolment and initiation on PrEP.

### Follow-up process

After enrolment, participants were seen at the clinic for follow-up six times during the 16-month demonstration project, with visits at month: 1, 3, 6, 9, 12, and 15. At each follow-up visit (quarterly), the clinical team saw participants for clinical tests for HIV, pregnancy, and creatinine. The counsellor then met with them to complete a follow-up questionnaire on their PrEP experience. In addition, STI screening using enhanced SCM approach, bi-annual cervical cancer screening using VIA, and syphilis screening using RPR were provided. At the exit visit (month 15), in addition to the questionnaire, all baseline tests were repeated. Participants who missed their follow-up visit date, could visit at a later time as an ‘unscheduled visit’. In addition to the routine tests done during the follow-up visits, study staff selected 85 participants for tenofovir blood level testing during their third month visit, with repeat testing done during their sixth month. Tenofovir testing was done in a lab in partnership with the National AIDS Research Institute (NARI).

At the field level, the project team designed a phased-out follow-up plan which included daily follow-up by peer outreach workers for the first 4–6 weeks and then streamlined to fortnightly over the following 4–6 weeks in order to match the TI guideline for outreach (as per the TI guideline, peer outreach workers are expected to meet their contacts in the field once every 15 days). Throughout the project, peer outreach workers tailored the frequency and types of support provided to meet the needs of participants (details of the peer outreach strategies will be presented in separate manuscripts). The community developed an adherence monitoring tool which was used by outreach workers to monitor adherence. PrEP was generally distributed once in 15 days. However, if participants were travelling out of the region, more pills might be provided to cover the travel period (usually not more than a one-month supply).

### Data entry and statistical analysis

The data entry team entered clinic forms and behavioural questionnaires from all visits in Epidata. Programme data (including screening, enrolment, follow-up visits, lab tests, and pill distribution details for each participant) was entered in MS Access. The data management team checked all data entered for consistency and correctness, then exported all data to Stata11 for data analysis. The data was declared panel-data by using ‘xtset’ command of Stata, with individual participant ID and the monthly visit time-point as the time variable. We present descriptive analysis on the changes in key outcomes.

### Ethics

The study received approval from the University of Manitoba, Ethics Review Board in Winnipeg, Canada, the Health Ministry Steering Committee (HMSC) in India, and the Institutional Ethics Review Board from DMSC, Kolkata, India. Informed consent was obtained from all participants in the study. All individuals had the right to refuse participation or were able to withdraw at any phases of the project.

## Results

**Figure 1.**
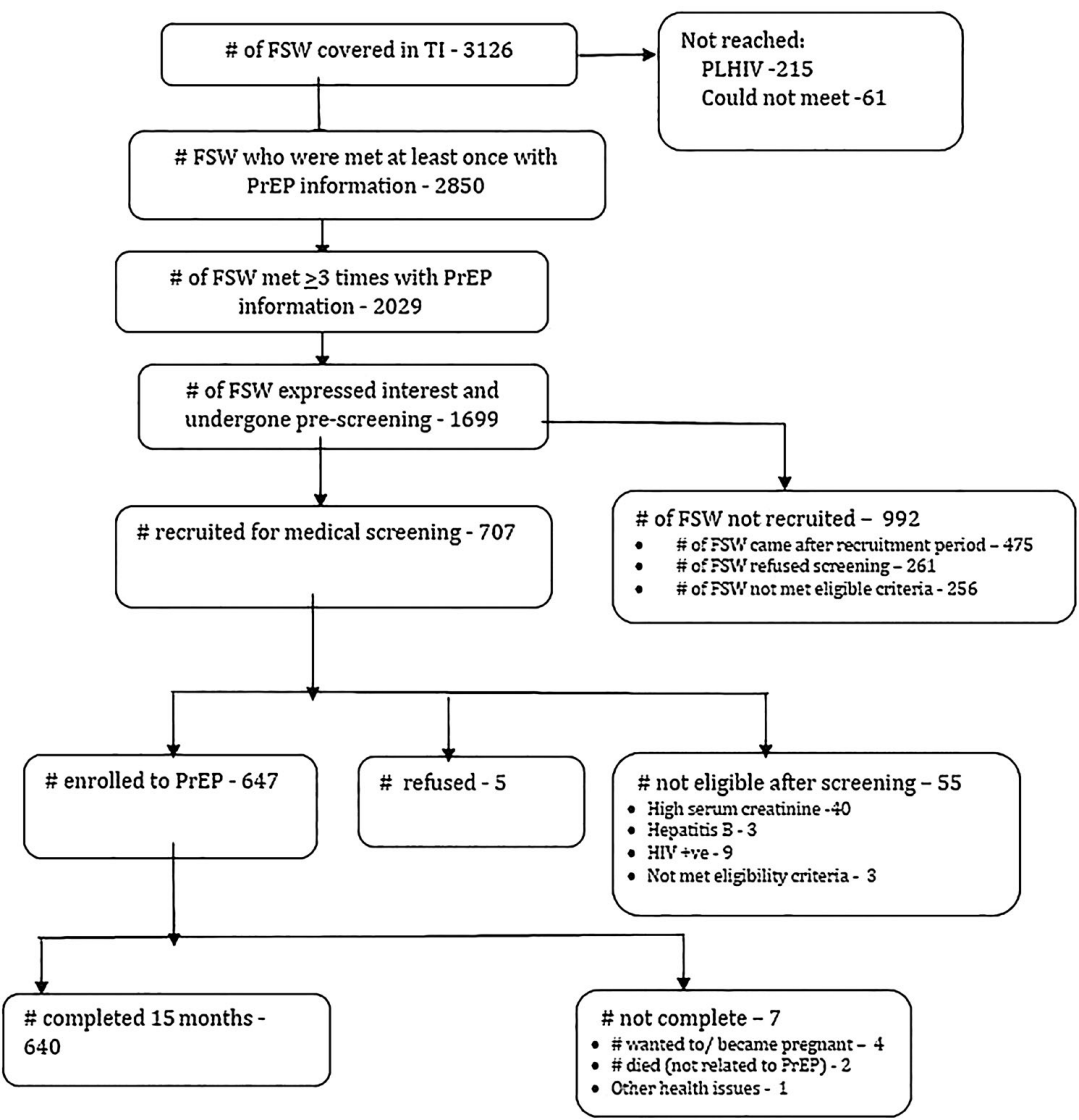
PrEP flow chart.

Ashodaya was working with 3126 female sex workers in Mysore and Mandya at the time the demonstration project was initiated. The peer outreach workers reached 2850 women at least once with PrEP-related information. Of those women, 2029 were reached ≥ 3 times and 1699 expressed interest in participating and went through the pre-screening assessment. Following pre-screening, 707 women were eligible to participate and recruited for medical eligibility screening during the recruitment period (March–October 2016). Of the remaining 992 women who were not recruited: 475 women, although eligible for recruitment, came to the clinic only after the recruitment phase was complete; 261 women requested more time to decide whether to participate and did not return within the recruitment phase; and 256 women were not eligible for participation. Among the 707 women who went through screening: 55 participants were ineligible due to: high serum creatinine (*n* = 40, 5.66%); testing positive for HIV (*n* = 9, 1.27%); testing positive for Hepatitis B (*n* = 3, 0.42%); or not meeting other eligibility criteria (*n* = 3, 0.42%). Of the 652 eligible participants, 5 FSWs refused to take part in the study after the screening process and 647 (99.23%) were enrolled. Of the 647 participants enrolled in the project, 7 exited from the study after enrolment (Figure 1).

### Socio-demographics and sex work characteristics

At the time of enrolment, the median age of participants was 35 years, 379 (58.58%) were illiterate and 333 (51.47%) were married. The median income during the last month was Rs.5400 (approx. $72.90USD) (Table 1). Only 92 (14.22%) sex workers worked in street-based settings, while the rest were working from homes and using cell phones for solicitation. Although at baseline, only 9 sex workers reported working away from their routine place of sex work, during the course of the project, many women reported to be away and therefore, took PrEP to cover their travel period. Of the 647 participants, 559 (86.40%) reported having regular partners. Of those with regular partners, only 134 (23.97%) knew the HIV status of their partners, with 8 partners (5.97%) reportedly living with HIV. Four of the partners (50%) living with HIV were on antiretroviral therapy (ART).

**Table 1.** Socio-demographic characteristics.

Characteristic	N (%)
N (all participants)	647
***Age (years)***	
Median (range)	35 (18–48)
<=20	5 (0.77)
21–25	43 (6.65)
26–30	150 (23.18)
31–35	151 (23.34)
>35	298 (46.06)
***Age at first commercial sex (years)***	
Median (range)	28 (15–44)
<=20	73 (11.28)
21–25	162 (25.04)
26–30	195 (30.14)
31–35	131 (20.25)
>35	86 (13.29)
***Number of years in sex work***	
Median (range)	6 (1–30)
<=5	309 (47.76)
6–10	242 (37.4)
11–15	61 (9.43)
>15	35 (5.41)
***Education***	
Illiterate	379 (58.58)
Literate	268 (41.42)
***Marital status***	
Never married	15 (2.32)
Married	333 (51.47)
Widow/divorced/separated	299 (46.21)
***Income (in rupees) during last month***	
Median (range)	5400 (2000–30000)
<=5000	322 (49.77)
>5000	325 (50.23)
***Living children***	
Median (range)	2 (0–6)
0 child	43 (6.65)
1–2 children	491 (75.89)
>2 children	113 (17.47)

### Follow-up visits and side effects

Follow-up visits were high, with 637 (98.45%) participants presenting for their follow-up appointment at the end of the 1st month, 642 (99.23%) participants presenting for their 3rd month visit, 641 (99.07%) women came for their 6th and 9th month visits, and 640 (98.92%) were at their 12th month and 15th month exit visits. At the end of their 1st month visit, 194 (30.46%) women complained of side effects, with complaints decreasing during subsequent visits. The most common side effects reported were nausea and vomiting which were immediately addressed by the physician and nurses. (Table 2).

**Table 2.** Follow-up visits and side effects.

Follow-up visit	Participants visited for follow-up (*N* = 647)	Participants experienced side effect n (%)
End of 1st month	637 (98.5%)	194 (30.46%)
End of 3rd month	642 (99.23%)	68 (10.59%)
End of 6th month	641 (99.07%)	38 (5.93%)
End of 9th month	641 (99.07%)	11 (1.72%)
End of 12th month	640 (98.98%)	0 (0%)
End of 15th month	640 (98.92%)	0 (0%)

### Clinical tests at follow-up visits

All PrEP follow-up visits to the clinic were integrated with the regular medical check-up (RMC) done as part of the TI programme. STI screening using enhanced SCM identified one case at the end of the 1st month follow-up, five cases at the end of 3rd month follow-up, and two cases at the end of 5th month follow-up. All cases were treated using the SCM approach. It is important to mention that there were no seroconversions for HIV among participants during the course of the demonstration project. There were also no reactive cases of syphilis. Cervical cancer screening did not detect any cervical lesions and serum creatinine levels remained within normal limits. Four participants became pregnant during the duration of the study and exited the project (Table 3).

**Table 3.** Clinical tests at follow-up visits.

Characteristic	End of 1st month		End of 3rd month		End of 6th month		End of 9th month		End of 12th month		End of 15th month (exit visit)	
	N	n(%)	N	n(%)	N	n(%)	N	n(%)	N	n(%)	N	n(%)
STI screening	637	1 (0.16)	642	5 (0.78)	641	2 (0.31)	641	0 (0)	640	0 (0)	640	0 (0)
HIV test –positive	637	0 (0)	642	0 (0)	641	0 (0)	641	0 (0)	640	0 (0)	640	0 (0)
Pregnancy Test- positive	637	0 (0)	642	2 (0.31)	641	0 (0)	641	1 (0.16)	640	0 (0)	640	0 (0)
Syphilis-reactive*	637	NA	642	NA	641	0 (0)	641	NA	640	0 (0)	640	NA
Cervical cancer screening-reactive*	637	NA	642	NA	641	0 (0)	641	NA	640	0 (0)	640	NA
Kidney function (high serum creatinine clearance)	637	0 (0)	642	0 (0)	641	0 (0)	641	0 (0)	640	0 (0)	640	0 (0)

*Syphillis and cervical cancer screening are done every six months.

### Condom use

**Figure 2.**
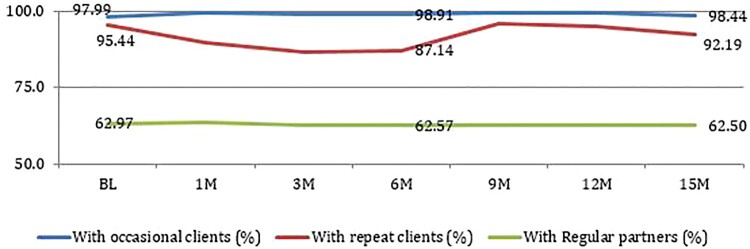
Condom use during last sex with clients and regular partners.

Self-reported condom use in the last sex with occasional clients and regular partners remained stable during the study period. Though condom use with regular clients decreased during the 1st, 3rd, and 6th month visits, it increased from the 9th month visit onwards and remained high (Table 4 and Figure 2). Condom use among regular partner remained stable throughout the study period.

**Table 4.** Condom use during last sex with clients and partners during last week.

Visit		Condom use with occasional clients (%)	Condom use with repeat clients (%)	Condom use with Regular partners (%)
Baseline	645	97.99	95.44	62.97
1st month	637	99.22	89.56	63.70
3rd month	642	98.91	86.50	62.70
6th month	641	98.91	87.14	62.57
9th month	641	99.22	95.91	62.57
12th month	640	99.22	94.97	62.50
15th month	640	98.44	92.19	62.50

### Adherence

**Figure 3.**
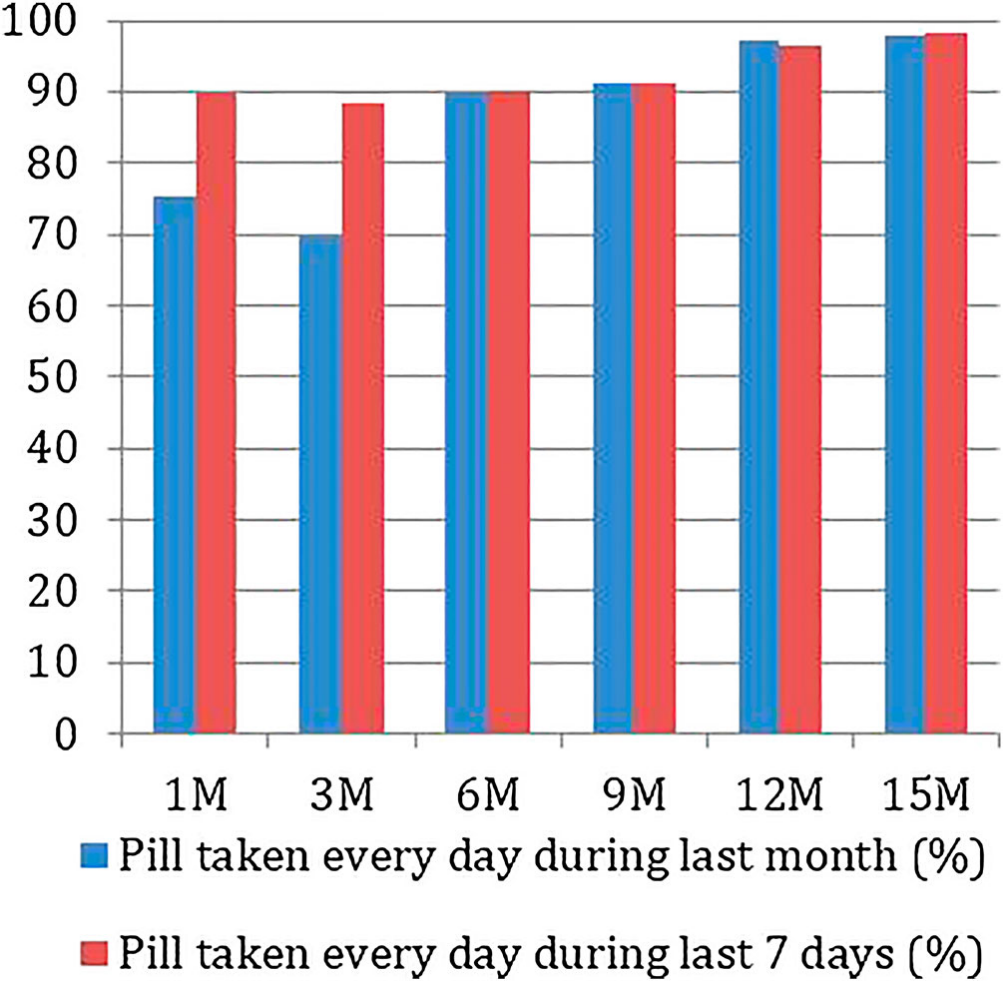
Self-reported adherence.

The project team measured adherence in three different ways. At the clinic visits, the counsellor recorded self-reported adherence in the follow-up questionnaires. Peer outreach workers tracked adherence on monitoring cards and through pill counts during PrEP distribution. In addition, a subset of participants (>10%) were tested for tenofovir blood level concentration. Self-reported daily adherence in the last month among participants was 75.35% at the end of the 1st month, but decreased to 69.63% in the 3rd month. At the end of 1st and 3rd month follow-up visit, 53 (8.32%) and 65 (10.12%) of participants reported to be non-adherent for the longest stretch of 7 days or more in the last one month. Subsequently, this reduced over time. Adherence increased in the 6th month to 89.70% and continued to improve until the study end, where participants reported a daily adherence of 97.97% in the last month (Table 5, Figure 3).

**Table 5.** Self-reported adherence.

Visit	N	Pill taken daily in last month n (%)	Pill taken daily in last 7 days n(%)	Longest stretch of non-adherence in last month (>=7 d) *n*(%)
1st month	**637**	480 (75.35)	573 (89.95)	53 (8.32)
3rd month	**642**	447 (69.63)	568 (88.47)	65 (10.12)
6th month	**641**	575 (89.70)	578 (90.17)	2 (0.31)
9th month	**641**	584 (91.11)	584 (91.11)	0 (0)
12th month	**640**	623 (97.34)	617 (96.41)	0 (0)
15th month	**640**	627 (97.97)	629 (98.28)	0 (0)

**Figure 4.**
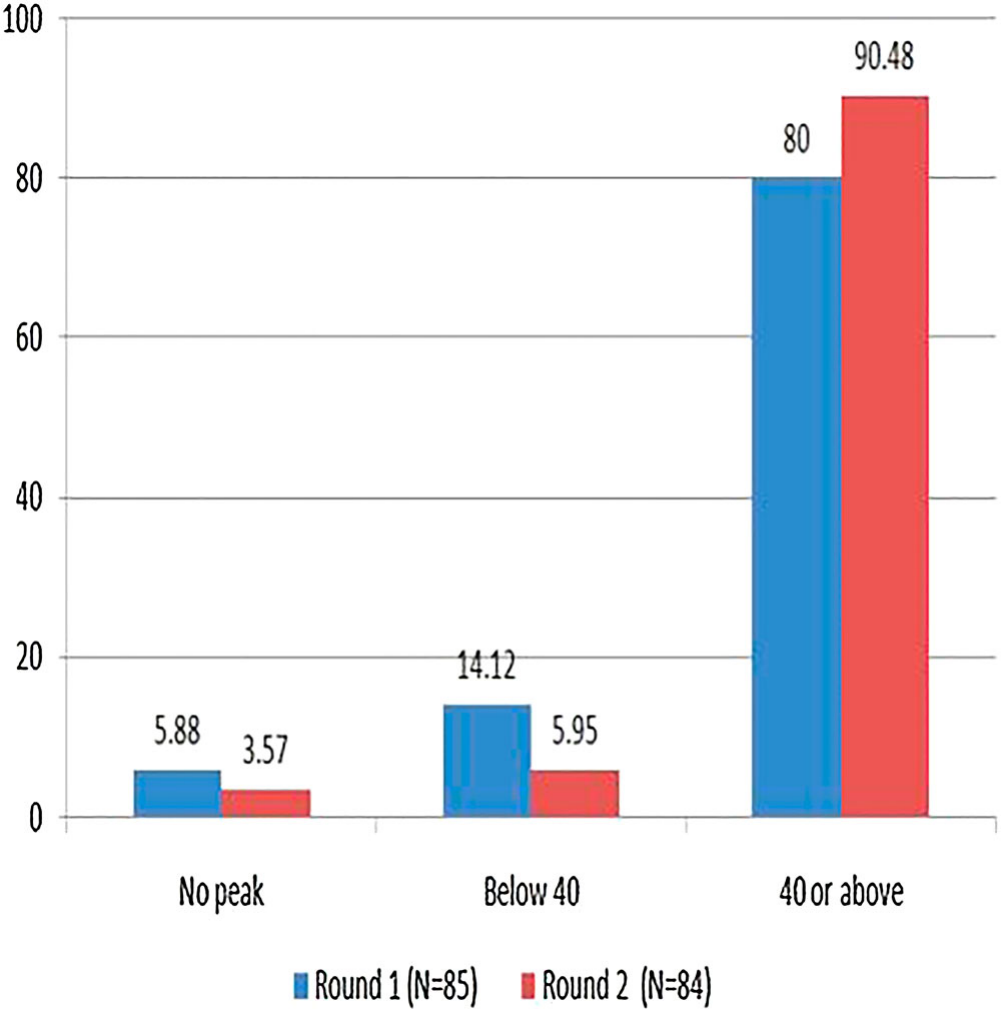
Round 1 and round 2 comparison for tenofovir concentration.

Tenofovir concentration was tested twice during the 16-month project, at the 3rd month and 6th month, among a subset of participants. A tenofovir concentration of 40 ng/mL was used as a marker for daily pill consumption in the last 7 days (Donnell et al., 2014). Of the participants tested, 80% (*n* = 68) in round 1 and 90.48% (*n* = 76) in round 2 had a tenofovir concentration of more than 40 ng/dL, indicating high adherence (Table 6, Figure 4).

**Table 6.** Round 1 and round 2 comparison for tenofovir concentration.

	Round 1	Round 2
Tenofovir Concentration (ng/mL)	n (%)	n(%)
No peak	5 (5.88)	3 (3.57)
Below 40	12 (14.12)	5 (5.95)
40 or above	68 (80)	76 (90.48)
Total	85	84

## Discussion

The Ashodaya PrEP Demonstration Project holds important insights for the roll out of PrEP within the context of the existing targeted interventions in India. Our retention and adherence rates are significantly higher than those reported in other demonstration project findings, pointing to the potential benefits of a community-led approach. Since Ashodaya, an established community-based organisation, led the study implementation and offered PrEP alongside their existing package of services, community leaders and peer outreach workers were able to successfully inform their peers about PrEP. This was done through a community preparation process that recruited a large number of participants into the study (*N* = 647) and provided support that led to high retention (98.92%) and high levels of reported adherence (97.97%). The intensive process of community preparedness and on-going engagement allowed sex workers to make informed decision on participation. While our feasibility study documented willingness and interest to take PrEP (Reza-Paul et al., 2016), the demonstration project documented high acceptability of PrEP. There were no HIV seroconversions, no increases in symptomatic STIs, and condom use with clients and partners in the last sex remained stable throughout the duration of the study. Providing information on PrEP through the system of peer outreach workers, fortnightly follow-up in the field, and quarterly clinic visits were points of integration with the existing TI programme.

Adherence was high in our study, while variations were observed at the start of the project. It is important to mention here that the study documented a dip in self-reported adherence around the third month, a period that corresponds to low or no clients because of demonetisation in India (Agarwal, 2018; Chari, 2016; Narayanan & Kohli, 2017). As in other demonstration projects with sex workers, we prioritised continued engagement over perfect adherence (Eakle et al., 2017). This allowed for periods of variable adherence, especially during the earlier months when people were getting used to taking PrEP. Drawing on Ashodaya’s existing network of peer outreach workers allowed for individualised adherence support strategies that best met the needs of participants, both in terms of scaling up support when dips in adherence were observed and through referrals to Ashodaya’s comprehensive package of health and social services beyond PrEP. Outreach strategies will be explored in separate manuscripts exploring these community-based processes (see for example, Lazarus, 2019).

Health care providers and researchers often flag risk compensation as a concern when discussing PrEP, however, as seen in our study, condom use remained consistent. This reflects findings in other studies, where PrEP clinical trials did not show substantial risk compensation (Marcus et al., 2013; Mugwanya et al., 2013). As stated by Mugo, Ngure, Kiragu, Irungu, and Kilonzo (2016) in a review of recent PrEP findings, evidence shows that PrEP uptake and adherence in demonstration projects seems to be driven by risk, that is, those who perceive themselves to be at risk are more likely to initiate PrEP. Similarly, Eakle et al. (2017) reported little change in condom use over time in their TAPS Demonstration Project, noting that women in PrEP studies may instead improve their attention to prevention strategies. Mathematical modelling looking at PrEP use among sex workers in South Africa has also shown that PrEP is likely to be of benefit in reducing HIV risk, even if reductions in condom use do occur (Grant et al., 2017). We also saw very few STIs in our study, pointing to the likelihood of continued consistent condom use. Side effects were not a major issue in our study, which is consistent with reports that PrEP is well tolerated (Eakle et al., 2017; Mayer & Ramjee, 2015; Mugo et al., 2016; Venter, 2018).

While our study took place within the context of a well-established community-led organisation, similar models of community mobilisation and support can be adopted to other environments. The Princess PrEP Program is another example of a community-led project among men who have sex with men and transgender women in Thailand, where community health workers who were already trained to provide peer-led HIV testing and counselling, received additional training to provide same-day PrEP in community health centre settings (Phanuphak et al., 2018). Community health workers provided visit reminders and continued adherence counselling that sought to address any challenges with adherence. While overall retention in the Princess Program was low (43.9% at 12 months), self-reported adherence was near perfect at 99.5% (Phanuphak et al., 2018).

Global roll out of PrEP has generally been slow, partly due to restricting delivery models to health facilities, as well as requirements around HIV testing and toxicity monitoring (Venter, 2018). Venter (2018), while addressing the challenges of scaling up PrEP, suggests that expanding access through specialised health services could be seen as an additional model for PrEP delivery. Shannon et al. (2018) echo that investments are needed in community and structural interventions for sex workers to benefit from PrEP. Our demonstration project provides important evidence that community-based sex work organisations can effectively deliver PrEP to their communities with extremely high levels of retention and adherence. When the World Health Organization and the Bill & Melinda Gates Foundation initiated discussions with Ashodaya sex worker community leaders regarding PrEP, these leaders initiated a series of community consultations which led to the feasibility study (Reza-Paul et al., 2016). Ashodaya leaders disseminated the feasibility study results to their community and further consultations were undertaken to determine the community-led design of the demonstration project. Through community consultations, Ashodaya decided that PrEP would be fully integrated into all of the components of their prevention programme, meaning that PrEP would be offered along with the organisation’s community-owned health services, outreach, and community mobilisation supports, making PrEP an integral part of the prevention intervention. Our study shows that community-delivered PrEP can be effectively integrated into the existing structures of HIV prevention and care services for sex workers and result in high retention and adherence.

Finding from some demonstration projects have led to PrEP being included in national HIV plans (Eakle et al., 2017). PrEP is not yet available through India’s National AIDS Control Organization’s HIV prevention programmes. Dissemination and advocacy is planned at the national level, as India prepares for its next National AIDS Control Prevention Strategy (NACP V). Our findings fit within calls to answer questions about the roll of community-led approaches to PrEP implementation and calls to increase community and sex worker-led involvement within biomedical interventions (Shannon et al., 2018).

### Limitations

Our study took place within a well-established community-led sex workers’ organisation and participants were recruited by peer outreach workers through their existing networks. Being exposed to a community-led intervention over time might predispose participants towards improved retention and adherence to PrEP. However, as we report in this study, having PrEP as an additional prevention tool in the existing TI programme led by a well-established community-based organisation holds important benefits for PrEP implementation. The design and protocol of the study did not take into consideration the cost effectiveness of PrEP within the context of the TI. Responses to surveys were self-reported, which could be influenced by social desirability bias, however, results from the tenofovir blood level testing support the high self-reported adherence. The findings presented in this paper rely on descriptive analysis on the changes in key outcomes. We have prepared a separate manuscript examining predictors of adherence to PrEP. It is critical to identify the predictors of retention, adherence, and condom use. All of the participants were linked to the existing TI programme; therefore, it is also important to understand if there are any differences in terms of condom use, number of symptomatic STIs, and pregnancy detection between the study participants and those who had not participated in the study, but are part of the TI programme. However, these analyses are beyond the scope of this manuscript and will be published separately.

### Conclusion

The Ashodaya PrEP Demonstration Project holds important insights for scaling up PrEP and highlights the importance of community-led programmes for ensuring high levels of PrEP adherence, which is a critical component for ensuring its efficacy in HIV prevention. We believe that the community-led models developed here hold lessons about community-led PrEP interventions that can be applied in other settings, by tapping into and trusting the knowledge held by community organisations to expand upon their existing package of HIV prevention services by adding on biomedical interventions. Following the demonstration project, Ashodaya is currently in the process of advocating for continued access to PrEP through inclusion of PrEP in India’s national HIV prevention guidelines.

## Data Availability

The datasets generated and/or analysed during the current study are not publicly available in order to protect the privacy and confidentiality of the study population. As Ashodaya Samithi is a community-based organisation led by the sex work community, and due to the criminalised nature of sex work in India and globally, the organisation has a policy of not making sensitive data sets publicly available in order to protect its members and ensure that members continue to feel safe participating in community-led research. To achieve access to the data used in this paper, interested scientists, clinicians, analysts, and researchers can contact Akram Pasha, Director of Ashodaya Academy, by email at ashodayasamithi@yahoo.co.in, who will then review and submit the application to access data to the Ashodaya Board. The Board will then review and respond to requests as appropriate.
